# Knowledge, Perception, and Prevention Practices Related to Human Papillomavirus-based Cervical Cancer and Its Socioeconomic Correlates Among Women in Karachi, Pakistan

**DOI:** 10.7759/cureus.7183

**Published:** 2020-03-05

**Authors:** Lubna Riaz, Sana Manazir, Fatima Jawed, Shajeea Arshad Ali, Ramsha Riaz

**Affiliations:** 1 Forensic Medicine and Toxicology, Dow Medical College, Dow University of Health Sciences (DUHS), Karachi, PAK; 2 Internal Medicine, Dow Medical College, Dow University of Health Sciences (DUHS), Karachi, PAK

**Keywords:** cervical cancer, socioeconomic, hpv, pap smear, vaccination, screening, pakistan, knowledge, prevention

## Abstract

Background

Cervical carcinoma is a widespread disease of the female genital tract, for which human papillomavirus (HPV) is an utmost risk factor. Of the total global burden, the majority is endured by the developing nations of the world, mainly due to inadequate knowledge regarding the disease and ineffective measures taken for its prevention, early detection, and screening. Hence, our study aimed to determine the level of knowledge, general attitudes and perception, and prevention practices related to HPV-based cervical cancer and its socioeconomic correlates among women in Karachi, Pakistan.

Methods

A cross-sectional, questionnaire-based study was conducted by approaching 450 females in the out-patient department (OPD) of a tertiary care hospital in Karachi, Pakistan from June 2019 to November 2019. The modified Kuppuswamy socioeconomic scale 2018 was deployed to assess the socioeconomic status of participants, while the knowledge score of the participants was determined based on the original Bloom's cut off point. The analysis was conducted using Statistical Package for Social Sciences (SPSS) version 24.0 (IBM Corp., Armonk, NY). Descriptive statistics were used to present the knowledge, attitude, and practice level of respondents. The respondents’ knowledge, attitude, and practice scores were compared across socioeconomic and demographic variables using the chi-square test.

Results

Of the 388 females interviewed, 199 (51.3%) were aware of the term cervical cancer, and 68 (34.2%) knew about Pap smear as a screening test; only 80 (40.2%) women were familiar with HPV vaccination as prophylaxis against cervical cancer. The practice of screening and prevention was found to be remarkably low (2.1% and 1.8% respectively). Socioeconomic status and education level had a significant association with knowledge of cervical cancer. Although around 64% of participants had poor knowledge, 308 (79.4%) demonstrated a favorable attitude as they were willing to know more about screening and preventive practices regarding cervical carcinoma.

Conclusion

The majority of the participants had insufficient overall knowledge about cervical cancer, HPV, Pap smear test, and HPV vaccination, highlighting the need for mass education through health professionals and media. In addition, the government authorities should provide screening services and vaccination against HPV free of cost to promote early detection of lesions and prophylaxis against this deadly disease.

## Introduction

The cervix is the lower end of the uterus, and cervical carcinoma is an abnormal rapid growth of anomalous cells of the cervix [1]. It is the second most frequent condition in females under 50 years of age and the fourth most prevalent cancer in women across all age groups worldwide [2-3]. In 2018, the World Health Organization (WHO) reported the incidence to be 570,000, constituting 7.5% of cancer-related female deaths, indicating that cervical carcinoma costs a life every two minutes all over the world [3-4]. South Asian countries constitute one-third of the total disease burden, while 85% of all deaths are reported in developing nations [3,5]. In Pakistan, the situation is even worse since it is one of the top ten countries with the highest female mortality rates, and 20 women are diagnosed with cervical cancer every day [6]. Moreover, the major age drift in the last few decades has increased incidence to 40% in the younger female population, with women aged 30-39 years being affected the most [5,7].

Cervical carcinoma may be asymptomatic in its early stages; nevertheless, patients can experience foul-smelling vaginal discharge and abnormal bleeding such as intermenstrual bleeding, post-coital bleeding, or postmenopausal bleeding [3]. The various risk factors that are linked to cervical cancer are high parity, early age at marriage, multiple sexual partners, smoking, low socioeconomic status, poor personal hygiene, and long-term estrogen exposure in the form of oral contraceptive pills [5]. Human papillomavirus (HPV) infection is found to be an essential causative factor in almost all cases of cervical carcinomas throughout the world, with 70% of the cases attributable to HPV oncogenic subtypes 16 and 18 [5]. It is quite alarming that more than one-third of all patients diagnosed with cervical carcinoma ultimately die when it is preventable and curable at an early stage, and around 50-90% of females who develop or die due to cervical cancer have never been screened [3,8-9]. 

Cervical cancer can be effectively controlled through primary, secondary, and tertiary preventive measures, which include prophylactic HPV vaccination, screening, diagnosis, and treatment of pre-cancerous and invasive cervical cancer [3]. Papanicolaou cytological testing (also known as Pap smear test) is used as a screening tool to identify precancerous lesions of the cervix, effectively lowering its incidence by 75-90% [10]. Although routine screening with Pap smear test has substantially reduced the incidence in the developed world, the scenario is entirely different in low- and middle-income countries, where dearth of screening facilities and HPV vaccination can be ascribed in part to lack of resources, but mainly to the serious lack of knowledge and attitude among population regarding early detection of cancer and its high mortality rates [5]. Likewise, the introduction of the HPV vaccine is limited by many factors such as its high cost [11]. Owing to these barriers, less than half of the participants of a study conducted among university students of Lahore, Pakistan knew about the prophylactic HPV vaccine [12]. Comparably, the awareness level among females in the most populated province of Pakistan was found to be low (29.9%) and surprisingly, only 37% of health professionals recognized Pap smear as a screening test [13-14]. This highlights that in addition to a lack of a mandatory screening practice in place for cervical cancer in our country, the poor knowledge and ineffective approach of healthcare professionals also act as major factors in contributing to the lack of awareness of the masses and subsequent poor prevention practices.

Socioeconomic status is highly associated with increased cervical cancer risk [15]. Hence, it is generally perceived that education level and socioeconomic conditions can influence the attitudes and awareness regarding risk factors and screening practices of females, which can eventually modify the potential of HPV-inducing cervical carcinoma. Thus, this study was conducted to assess the various variables that may affect the knowledge and perception of female population belonging to different socioeconomic settings of Karachi, Pakistan regarding cervical cancer, its major symptoms and risk factors, its relation to HPV, its early detection and prevention through screening and vaccination, and practice and attitude towards screening and prophylactic HPV vaccination.

## Materials and methods

A descriptive, cross-sectional study was conducted in the waiting area of the out-patient department (OPD) of a tertiary care hospital in Karachi, Pakistan between June 2019 and November 2019. The study population consisted of females aged between 17-65 years and were recruited using the convenience sampling technique. Questionnaire-based interviews were conducted after obtaining informed consent from all participants, and their confidentiality and anonymity were maintained. Females with a history of cervical cancer, females with any gynecological disease, and those who did not give consent were excluded from the study. The sample size was calculated through the OpenEpi sample size calculator [16]. Keeping a confidence interval (CI) of 95% and a 5% degree of precision, the estimated sample size was 317 using an anticipated frequency (p) of 29.1% [13]. In order to get the maximum response, we increased sample size and approached 450 females, out of which 396 consented to be part of the study. After excluding incomplete questionnaires, the response rate was found to be 86.22%.

The questionnaire was formulated based on objectives of the study after going through the relevant data available on the topic [4,9,12-14]. The questionnaire was pretested on 30 females before the survey to validate and modify the questions accordingly. The final questionnaire was divided into four main sections. The first section inquired about the socio-demographic data of respondents. The second section consisted of 22 items and was further divided into six sub-sections that assessed the knowledge regarding cervical cancer, its symptoms, its risk factors, HPV, cervical cancer screening, and prevention respectively. The third and fourth sections of the questionnaire assessed the attitudes and practices of respondents related to cervical cancer screening and prevention. The attitude was assessed by asking if they would be willing to get a Pap smear test and HPV vaccination. They were also inquired whether they would be interested to know more about the screening and prevention of cervical cancer. Practices were assessed by inquiring if they had undergone the Pap smear test and if they were vaccinated against HPV.

The modified Kuppuswamy socioeconomic scale updated for the year 2018 was used to assess the socioeconomic status of respondents [17]. Family incomes mentioned in the modified Kuppuswamy socioeconomic scale in Indian rupees were converted to Pakistani rupees (PKR) using an online converter and were rounded off to the nearest 500 in PKR [18]. Using relevant data, respondents were classified according to this scale as upper (i.e. upper), upper-middle and lower-middle (i.e. middle), upper-lower (i.e. poor) and lower (i.e. very poor) classes.

A 30-point scale was used to assess the knowledge of cervical cancer. Each sub-section of knowledge was assigned five points so that respondents were expected to score between 0-30 points. Every correct answer was assigned one point and wrong answer zero point. Only those respondents who answered "Yes" to the first question of the questionnaire, “Do you know about cervical cancer?”, were given a knowledge score. While evaluating the knowledge score, the knowledge level was based on the original Bloom's cut off points [19]. Respondents who scored between 24-30 points were considered as having good knowledge with 80-100% correct responses, those who scored between 18-23 points as having moderate knowledge with 60-79% correct responses, and those who scored <18 points as having poor knowledge with <60% correct responses. Knowledge scores for each sub-section were further grouped on the basis of three or more correct answers as “good” and two or less than two correct answers as “bad” scores respectively. Those participants who answered “yes” in at least two out of three attitude questions were considered as having a positive attitude, while those who said no in two out of three questions were categorized as having a negative attitude. Practices were analyzed by classifying respondents as having “regular practice” for those who have had Pap smear test and vaccination both done or any one of them done, and “irregular practice” for those who have had neither Pap smear test nor vaccination done.

Data were analyzed using Statistical Package for Social Sciences (SPSS) version 24.0, (IBM Corp., Armonk, NY). Descriptive statistics, e.g., frequencies, percentage, and mean and standard deviation were used for categorical and continuous variables respectively. The chi-square test was performed to determine the association between socioeconomic and demographic variables with knowledge, attitude, and practice (KAP) levels. A p-value of <0.05 was considered statistically significant.

## Results

Table 1 outlines the socio-demographic parameters of 388 participants included in the study. The mean age of the females was 33.59 ±13.36 years (range: 17-65 years). Of the 388 participants, 238 (61.3%) were married at some point in their lives. The age range of females at the time of marriage was 17-38 years with 20 being the most common age. The participants were distributed in different socioeconomic classes according to the modified Kuppuswammy socioeconomic status scale 2018 as shown in Table 1 [17].

**Table 1:** Socio-demographic parameters of the respondents (n = 388) SD: standard deviation ^a^Based on modified Kuppuswammy socioeconomic status scale 2018

		N (%) ±SD
Age, years	17-26	155 (39.9) ±13.4
	27-36	87 (22.4) ±13.4
	37-46	68 (17.5) ±13.4
	47-56	50 (12.9) ±13.4
	Above 56	28 (7.2) ±13.4
Marital status	Single	150 (38.7)
	Married	197 (50.8)
	Divorced/separated	18 (4.6)
	Widowed	23 (5.9)
Number of children	0	35 (14.7)
	1-2	72 (30.3)
	3-5	90 (37.8)
	>5	41 (17.2)
Education	No education	54 (13.9)
	Primary school	39 (10.1)
	Secondary school	64 (16.5)
	College	97 (25.0)
	University	134 (34.5)
Occupation	Student	103 (26.5)
	Housewife/not working	185 (47.7)
	Working	93 (24.0)
	Jobless/retired	7 (1.8)
Socioeconomic status^a^	Upper class (I)	32 (8.2)
	Upper middle class (II)	101 (26.0)
	Lower middle class (III)	75 (19.3)
	Upper lower class (IV)	98 (25.3)
	Lower class (V)	82 (21.1)

Among the 388 females interviewed, only 199 (51.3%) were aware of the term cervical cancer. These 199 females were further interviewed to test their knowledge related to HPV and epidemiology, screening, and prevention of cervical cancer as shown in Table 2.

**Table 2:** Knowledge regarding epidemiology, screening, and prevention of cervical cancer and human papillomavirus HPV: human papillomavirus; Pap: Papanicolaou

	Yes, n (%)	No, n (%)	Don’t know, n (%)
Knowledge regarding epidemiology of cervical cancer			
Is cervical cancer one of the most common cancers among females?	81 (40.7)	47 (23.6)	71 (35.7)
Are all women at risk of developing cervical cancer?	59 (29.6)	77 (38.7)	63 (31.7)
Is cervical cancer more common in middle-aged (35-50 years) females?	76 (38.2)	48 (24.1)	75 (37.7)
Is cervical cancer a communicable disease (transmitted by skin contact, sneezing, coughing)?	53 (26.6)	100 (50.3)	46 (23.1)
Knowledge regarding HPV			
Is HPV infection rare in Pakistan?	53 (26.6)	64 (32.2)	82 (41.2)
Can HPV infection affect both males and females?	97 (48.7)	35 (17.6)	67 (33.7)
Is HPV transmitted by sexual contact?	108 (54.3)	16 (8.0)	75 (37.7)
Can HPV cause cancers other than cervical cancer?	72 (36.2)	16 (8.0)	111 (55.8)
Can HPV cause genital warts?	70 (35.2)	4 (2.0)	125 (62.8)
Knowledge regarding screening of cervical cancer			
Is there any test available for screening of cervical cancer?	119 (59.8)	4 (2.0)	76 (38.2)
Is Pap smear a screening test for cervical cancer?	68 (34.2)	5 (2.5)	126 (63.3)
Should screening be initiated at 25 years of age in every female even if asymptomatic?	68 (34.2)	37 (18.6)	94 (47.2)
Is screening done for sexually active people only?	68 (34.2)	49 (24.6)	82 (41.2)
Should screening be repeated every three years in every female even if asymptomatic?	56 (28.1)	31 (15.6)	112 (56.3)
Knowledge regarding prevention of cervical cancer			
Does HPV vaccine prevent cervical cancer?	80 (40.2)	13 (6.5)	106 (53.3)
Is HPV vaccine given to males and females both?	79 (39.7)	29 (14.6)	91 (45.7)
Should HPV vaccine be given before 26 years of age?	51 (25.6)	29 (14.6)	119 (59.8)
Should HPV vaccine be given to sexually active people only?	61 (30.7)	45 (22.6)	93 (46.7)
Is there any need for screening even after HPV vaccination?	79 (39.7)	20 (10.0)	100 (50.3)

**Figure 1:**
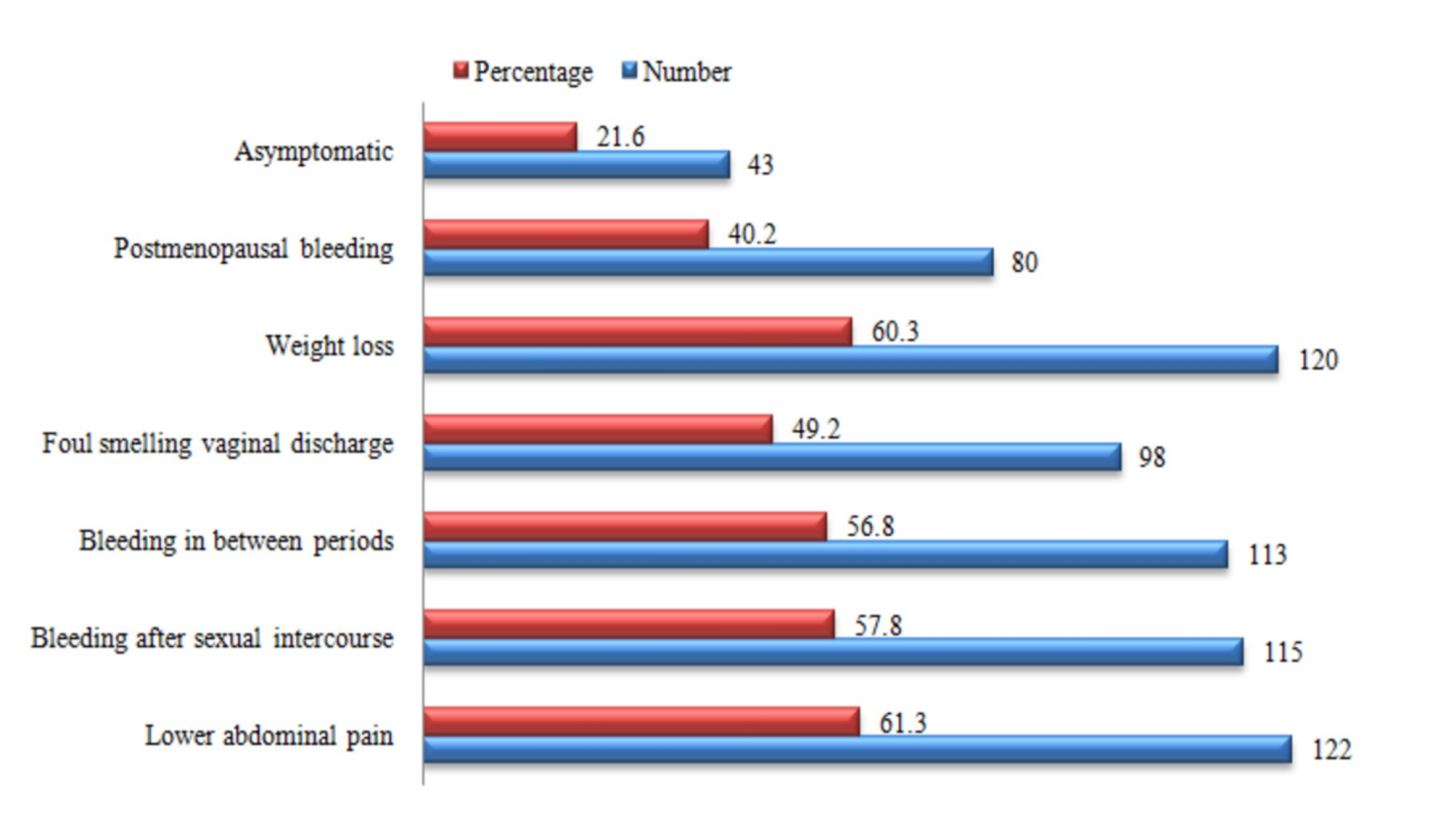
Knowledge of the participants regarding symptoms of cervical cancer

**Figure 2:**
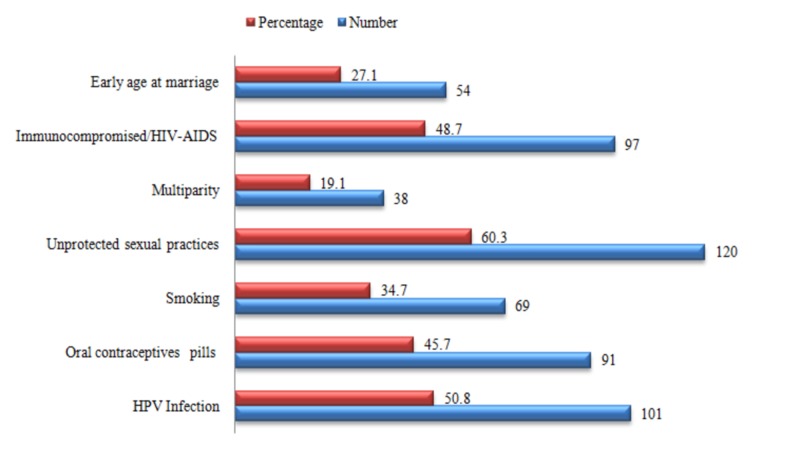
Knowledge of the participants regarding risk factors of cervical cancer HIV: human immunodeficiency virus; AIDS: acquired immunodeficiency syndrome; HPV: human papillomavirus

Those 199 females were also questioned regarding their knowledge of symptoms and risk factors associated with cervical cancer (Figures 1-2). Most of the women were aware of lower abdominal pain and weight loss as being the predominant symptoms of cervical cancer. Similarly, a preponderance among the responses regarding the risk factors “unprotected sexual practices” and “HPV infection” was observed.

**Figure 3:**
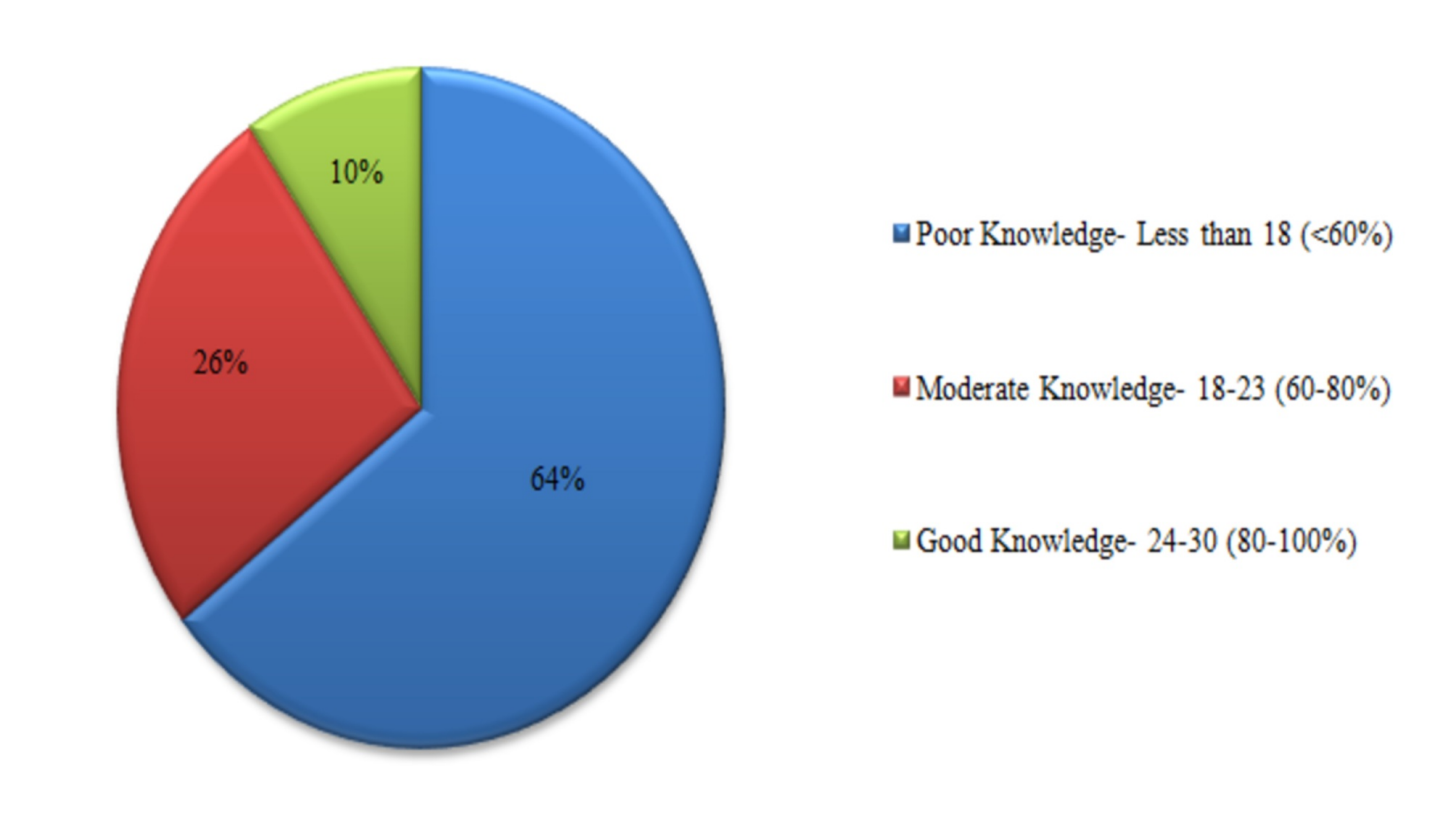
Knowledge score of the participants based on the original Bloom's cut-off point

The participants of the study were scored in each sub-section based on their knowledge regarding HPV and the epidemiology, screening, prevention, risk factors, and symptoms of cervical cancer; 49.2% of the people obtained a good score regarding knowledge of cervical cancer, 64.8% for symptoms, 55.8% for risk factors, 40.2% regarding HPV, 28.6% for screening, and 36.2% had a good score regarding prevention of cervical cancer. The total knowledge score that each participant obtained in all sub-sections was added up and classified based on the original Bloom’s cut-off point (Figure 3).

**Figure 4:**
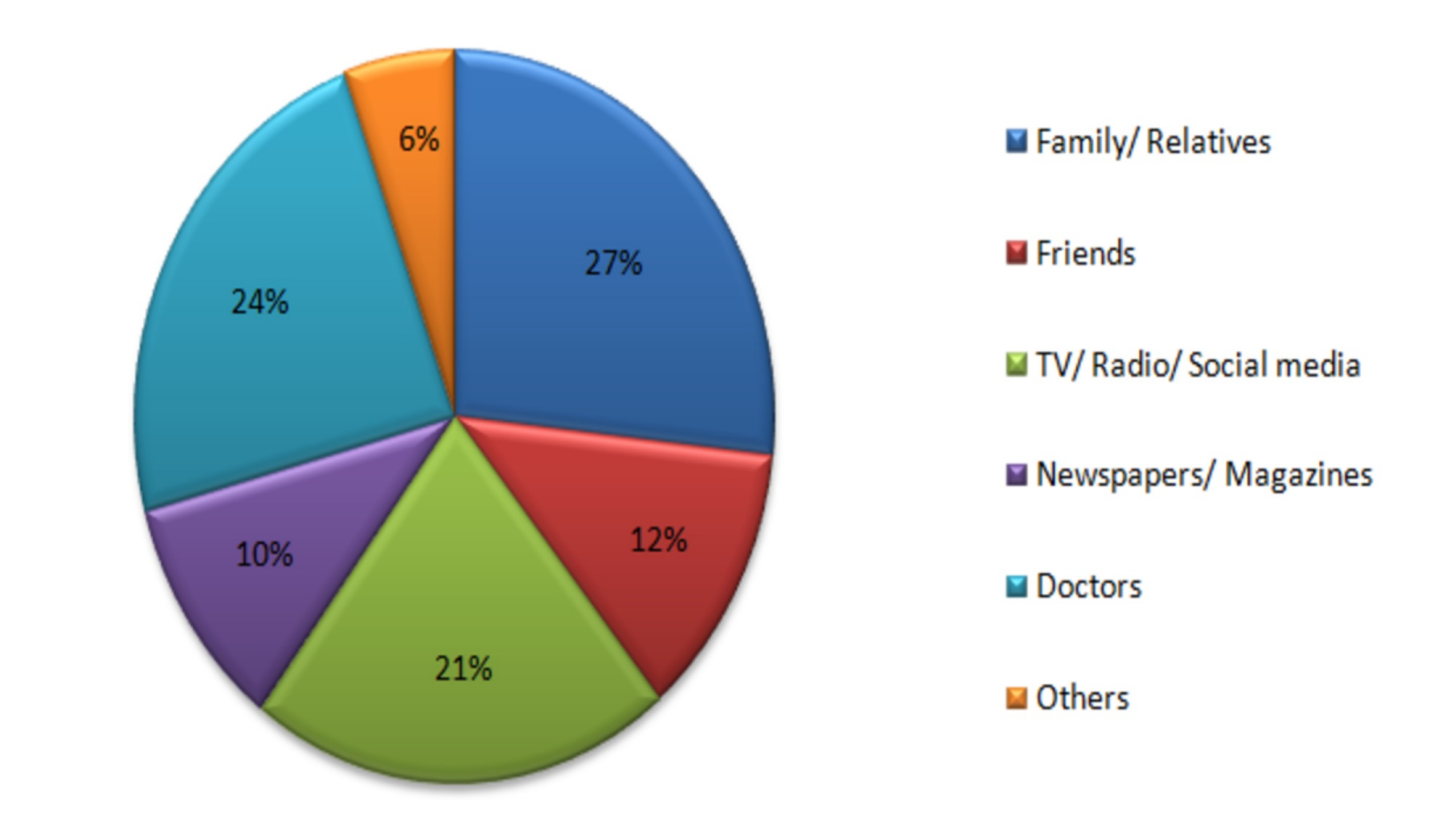
Source of information about cervical cancer

The participants were enquired about the sources of information regarding cervical cancer and the major sources were found to be family/relatives, doctors, and TV/radio/social media respectively (Figure 4).

All women (388) were interviewed to assess their willingness to undergo screening and undertake prevention practices regarding cervical cancer. In our study, the majority of participants had a negative attitude towards HPV vaccination and Pap smear test, whereas a majority was eager to know more about the screening and prevention practices (Table 3).

**Table 3:** Attitude towards cervical cancer screening and prevention Pap: Papanicolaou

	Yes, n (%)	No, n (%)
Would you go for a Pap smear test now?	117 (30.2)	271 (69.8)
Do you intend to get vaccinated now?	145 (37.4)	243 (62.6)
If no: will you consider getting vaccinated if the government provides it free of cost?	118 (48.6)	125 (51.4)
Would you like to know more about screening and prevention of cervical cancer?	308 (79.4)	80 (20.6)

**Figure 5:**
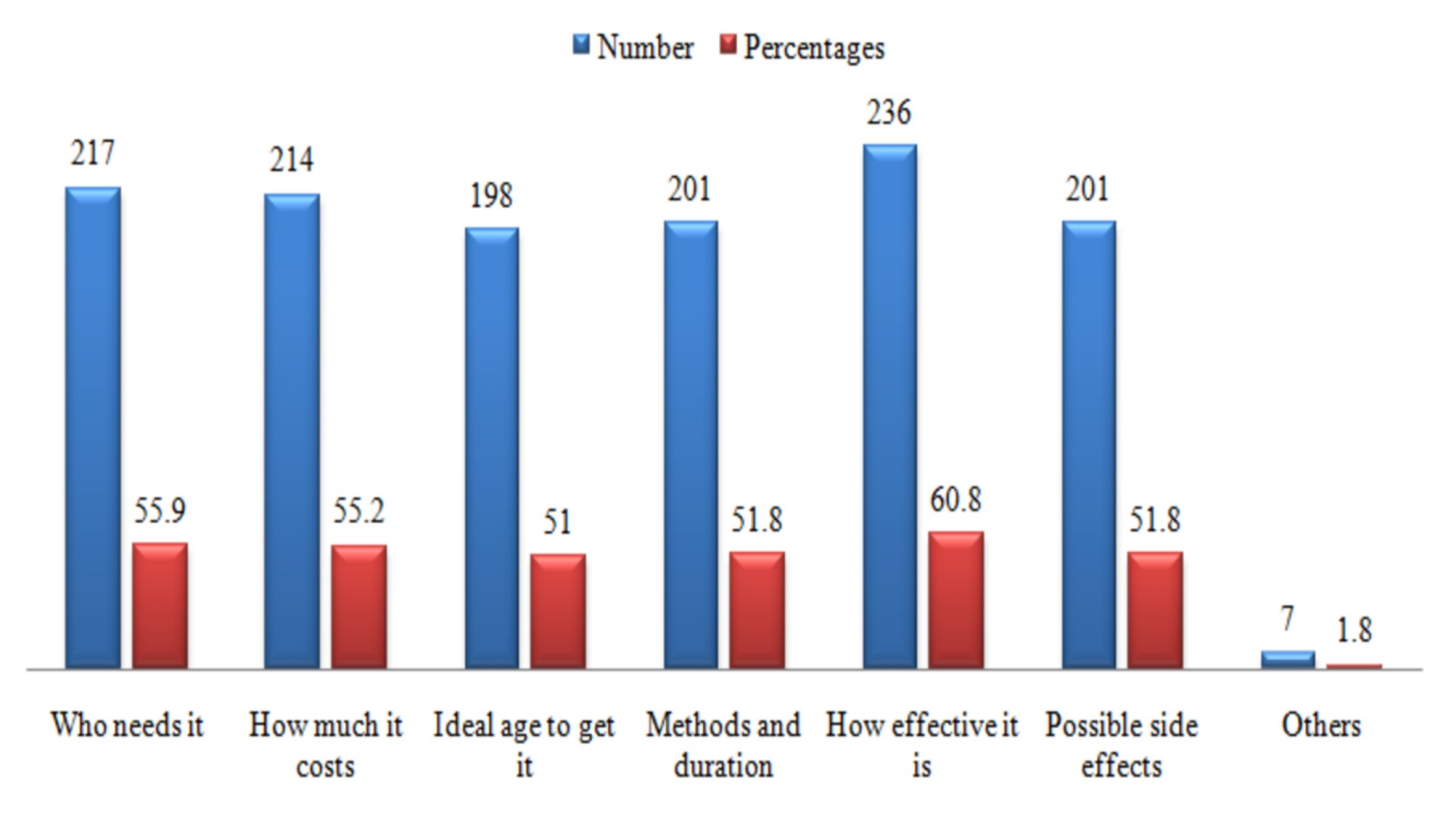
Information regarding screening and prevention of cervical cancer that the respondents wanted to know

The respondents were further interviewed regarding what they would like to know more about screening and prevention practices of cervical cancer. Most of the population was inclined to know more about the efficacy of these practices (Figure 5).

The participants of the study were questioned regarding their practices related to screening and prevention of cervical cancer. The majority were seen to observe “bad practices” as only eight people out of 388 had a Pap smear test done during their lifetime, and only seven were found to be vaccinated against HPV. The reasons for these unsatisfactory practices were asked, and the most prevalent was the lack of awareness regarding these practices (Table 4).

**Table 4:** Practices regarding cervical cancer screening and prevention Pap: Papanicolaou; HPV: human papillomavirus

		N (%)
Have you ever had a Pap smear done?	Yes	8 (2.1)
	No	380 (97.9)
If yes, when was the last time you had a Pap smear?	Less than 1 year ago	1 (12.5)
	Less than 2 years ago	2 (25.0)
	Less than 3 years ago	2 (25.0)
	More than 3 years ago	3 (37.5)
If no, what is the reason?	Don't know about the test	226 (59.5)
	I don't need it right now	85 (22.4)
	Expensive	58 (15.3)
	Might be painful	35 (9.2)
	Time-consuming	31 (8.2)
	Embarrassing	28 (7.4)
	Parents/spouse won't allow	14 (3.7)
	Fear of being diagnosed with cancer	10 (2.6)
	Others	4 (1.1)
Are you vaccinated against HPV?	Yes	7 (1.8)
	No	381 (98.2)
If no, what is the reason?	Lack of knowledge	224 (58.8)
	I don't need it right now	113 (29.7)
	Expensive	61 (16.0)
	Might be painful	37 (9.7)
	Worry about side effects	35 (9.2)
	Time-consuming and requires multiple doses	35 (9.2)
	Doubts on vaccine efficacy	23 (6.0)
	Parents/spouse won't allow	18 (4.7)
	Embarrassing to receive a sexually transmitted infection (STI) vaccine	11 (2.9)
	Others	3 (0.8)

Table 5 shows the assessment of knowledge based on participants' level of education and their socioeconomic status. There was a statistically significant relationship between the socioeconomic status of participants and their knowledge regarding the term 'cervical cancer', whether it is a communicable disease, Pap smear test, and HPV vaccine. Participants belonging to the upper class were observed to have better knowledge as compared to those in other socioeconomic classes. Similarly, females who were university graduates were observed to have better knowledge than those with comparatively lower levels of education.

**Table 5:** Assessment of knowledge according to the modified Kuppuswamy socioeconomic status scale 2018 and education level Pap: Papanicolaou; HPV: human papillomavirus ^a^Calculated using chi-square for categorical data; a p-value of <0.05 considered statistically significant

Modified Kuppuswamy socioeconomic status scale 2018	Upper class (I), n (%)	Upper middle class (II), n (%)	Lower middle class (III), n (%)	Upper lower class (IV), n (%)	Lower class (V), n (%)	P-value^a^
Do you know about the term “cervical cancer”?	23 (71.9)	69 (68.3)	49 (65.3)	44 (44.9)	14 (17.1)	0.000
Is cervical cancer a communicable disease?	18 (78.3)	55 (79.7)	37 (75.5)	31 (70.5)	5 (35.7)	0.016
Is Pap smear a screening test for cervical cancer?	15 (65.2)	31 (44.9)	13 (26.5)	7 (15.9)	2 (14.3)	0.000
Does HPV vaccine prevent cervical cancer?	19 (82.6)	36 (52.2)	14 (28.6)	10 (22.7)	1 (7.1)	0.000
Education level	No education, n (%)	Primary school, n (%)	Secondary school, n (%)	College, n (%)	University, n (%)	
Do you know about term “cervical cancer”?	28 (51.9)	16 (41.0)	25 (39.1)	42 (43.3)	88 (65.7)	0.001
Is cervical cancer a communicable disease?	11 (39.3)	10 (62.5)	17 (68.0)	34 (81.0)	74 (84.1)	0.000
Is Pap smear a screening test for cervical cancer?	4 (14.3)	2 (12.5)	3 (12.0)	15 (35.7)	44 (50.0)	0.000
Does HPV vaccine prevent cervical cancer?	4 (14.3)	3 (18.8)	6 (24.0)	19 (45.2)	48 (54.5)	0.000

We observed that there was a significant association between the participants’ age, marital status, education level and positive attitude regarding cervical cancer screening and prevention (Table 6). Participants, who were of younger age group, were married, and those who had higher education were observed to have a positive attitude than their counterparts. There was no statistically significant relationship found between positive attitude and socioeconomic status of participants. This table also reveals that there was a statistically significant relationship observed between positive attitude and good knowledge scores regarding HPV, cervical cancer, and its symptoms and screening.

**Table 6:** Assessment of attitude regarding cervical cancer screening and prevention according to the socio-demographic parameters and knowledge scores HPV: human papillomavirus ^a^Calculated using chi-square for categorical data; p-value of <0.05 considered statistically significant ^b^Based on modified Kuppuswammy socioeconomic status scale 2018

		Positive attitude, n (%)	Negative attitude, n (%)	P-value^a^
Age, years	17-26	71 (45.8)	84 (54.2)	0.004
	27-36	54 (62.1)	33 (37.9)	
	37-46	48 (70.6)	20 (29.4)	
	47-56	28 (56.0)	22 (44.0)	
	Above 56	12 (42.9)	16 (57.1)	
Marital status	Single	72 (48.0)	78 (52.0)	0.028
	Married	120 (60.9)	77 (39.1)	
	Divorced/separated	12 (66.7)	6 (33.3)	
	Widowed	9 (39.1)	14 (60.9)	
Education level	No education	21 (38.9)	33 (61.1)	0.045
	Primary school	25 (64.1)	14 (35.9)	
	Secondary school	40 (62.5)	24 (37.5)	
	College	49 (50.5)	48 (49.5)	
	University	78 (58.2)	56 (41.8)	
Socioeconomic status^b^	Upper class (I)	21 (65.6)	11 (34.4)	0.379
	Upper middle class (II)	57 (56.4)	44 (43.6)	
	Lower middle class (III)	45 (60.0)	30 (40.0)	
	Upper lower class (IV)	48 (49.0)	50 (51.0)	
	Lower class (V)	42 (51.2)	40 (48.8)	
Knowledge about cervical cancer	Good score	74 (60.2)	24 (31.6)	0.000
	Bad score	49 (39.8)	52 (68.4)	
Knowledge about symptoms	Good score	89 (72.4)	40 (52.6)	0.005
	Bad score	34 (27.6)	36 (47.4)	
Knowledge about risk factors	Good score	72 (58.5)	39 (51.3)	0.319
	Bad score	51 (41.5)	37 (48.7)	
Knowledge about HPV	Good score	58 (47.2)	22 (28.9)	0.011
	Bad score	65 (52.8)	54 (71.1)	
Knowledge about screening	Good score	42 (34.1)	15 (19.7)	0.029
	Bad score	81 (65.9)	61 (80.3)	
Knowledge about prevention	Good score	50 (40.7)	22 (28.9)	0.095
	Bad score	73 (59.3)	54 (71.1)	

## Discussion

Cervical cancer is one of the most preventable cancers among women [13]. Despite that, it is the third leading cause of mortality among the female population worldwide, especially in countries with stringent healthcare budgets, such as Pakistan [13]. This can be attributed to the lack of outreach by the government and media in order to raise awareness about cervical cancer. National screening and vaccination programs in developed countries such as Australia and the United Kingdom have reduced cervical cancer burden up to 90% [12,20]. However, in developing countries such as Pakistan, there is a dearth of such resources and taboos associated with this disease. Owing to this, women are unable to undertake proper measures towards screening and prevention of this deadly disease, contributing to the huge mortality rates associated with cervical cancer [12,21]. This study aimed to assess the knowledge of the general population about cervical cancer epidemiology, etiology, risk factors, symptoms, and their attitudes and practices related to screening and prevention. Moreover, the association of socio-demographic parameters, especially economic stability, with knowledge and attitude was investigated as there was a positive correlation observed in previous studies conducted globally [20,22-23].

Our study has highlighted an overall lack of knowledge regarding HPV and the epidemiology, screening, prevention, risk factors, and symptoms of cervical cancer, with 64% of the respondents securing a total knowledge score of less than 60% and only 10% being able to score above 80%. A review by Raychaudhuri and Mandal shows that the same situation prevails worldwide as studies conducted in high-income countries like Japan and Korea emphasize a lack of awareness regarding the disease [5]. Studies conducted in low- and middle-income countries like South Africa, Ethiopia, and Indonesia also demonstrated subpar knowledge [5,24]. Furthermore, only 25.5% of women obtained a poor knowledge score in the neighboring country India, which is substantially better than the findings of our study, underlining the status of substandard knowledge among women in Pakistan [19].

The current study depicted that only half of the women visiting the tertiary care hospital of the most populated city of Pakistan were aware of the term “cervical cancer.” This finding contrasts with the studies conducted in the Democratic Republic of Congo and India where the awareness regarding cervical cancer was much higher [19,25]. On the contrary, another study carried out in Pakistan showed that the awareness level of women was below average [13]. Similarly, a survey done in Karachi among health workers revealed that only 1.8% of the participants did not know about cervical cancer as a disease [14]. This depicts that there is an evident gap of knowledge between the general population and healthcare workers, which needs to be bridged.

Our study showed that the awareness regarding signs and symptoms of cervical cancer and risk factors associated with it was only 34.2% and 19.6% respectively among the participants, which was drastically below par when compared with the regional study of Narayana G et al. where twice as many women were familiar with the presenting complains and risk factors respectively (Table 7) [19]. This lack of knowledge usually contributes to the high mortality associated with cervical cancer because women do not take proper preventive measures if they are unenlightened about the risk factors. Likewise, late presentation or failure of recognition of the symptoms may lead to delayed seeking of medical attention, leading to poor prognosis and increased mortality.

**Table 7:** Comparison between current study results and results of similar studies on knowledge, attitude, and practices regarding cervical cancer Pap: Papanicolaou; HPV: human papillomavirus

	Ali-Risasi et al. [25], %	Khan et al. [13], %	Narayana et al. [19], %	Current study, %
Aware of the term “cervical cancer”	81.9	29.1	74.6	51.3
Aware of Pap smear test	16.8	18.1	2.0	34.2
Aware of HPV vaccine			74.7	40.2
Knowledge about risk factors of cervical cancer			62.8	55.8
Knowledge about symptoms of cervical cancer			64.2	64.8
Positive attitude towards screening test for cervical cancer	79.6			30.2
Good practice regarding screening of cervical cancer	8.6		13.4	2.1
Good practice regarding prevention of cervical cancer		14.9		1.8

In our population, 40.7% of women were aware that cervical cancer was one of the most common gynecological cancers in contrast with the knowledge of healthcare workers of Pakistan reported in a study, in which only one-fourth of the participants were found to be aware of the seriousness and virulence of this disease [14]. Despite being aware of the prevalence of this disease, only 54.3% knew that HPV was transmitted via sexual contact. This is similar to findings by a study conducted in Lahore, Pakistan where only about half of the population was aware of the sexually transmitted nature of HPV [12]. This can be attributed to the social and cultural taboos associated with sexually transmitted diseases (STDs), which means that information is not widely shared due to the stigma surrounding this topic and, hence, ignorance prevails [13]. Furthermore, among those who had heard of cervical cancer, around one-fourth presumed it to be a communicable disease, which is comparable to a study conducted in the Maldives where 15.5% believed the disease to be infectious in nature [26]. This depicts a lack of knowledge regarding the etiology of the disease and can potentially further stigmatize the disease, causing isolation and preventing women from seeking medical treatment [26].

Furthermore, in our study, only 34.2% were aware that Pap smear is a screening test for cervical cancer, and only 2.1% had gone for Pap smear test during their lifetime, whereas in Nigeria, another developing country, 55.1% were aware of the Pap smear test and 22.9% had undergone this screening test [27]. The lack of knowledge and unsatisfactory practices were not unexpected in our study given that a recent research study carried out in Pakistan showed that only 35.4% had heard about the Pap smear test and only 5.9% had opted to go for it [21]. A study by Imam et al. conducted in Lahore, Pakistan reported that about 95% of women were never advised to go for a Pap smear test by any doctor [28]. Similarly, one of the main barriers towards the Pap smear test was the non-recommendation by physicians as found in studies conducted in Nigeria, Kuwait, and Iran [27]. Hence, it is evident that cervical cancer will continue to impose a toll globally unless health workers counsel women regarding the available screening and treatment options. Moreover, there has been a significant reduction in the rates of cervical cancer morbidity and mortality in the United States (US), Canada, and the majority of European countries due to the highly efficient screening programs put into place [5]. Whereas, developing countries like Pakistan are lagging behind and will continue to bear the burden of this disease unless they establish such national screening programs.

Prevention of HPV infections is essential to bring down the prevalence of cervical cancer, which is possible by the use of HPV vaccination; therefore, this important preventive tool should be made easily available to the general population. However, our study found that only 40.2% were aware of HPV vaccine's ability to prevent cervical cancer and only 1.8% were vaccinated against it, which is similar to the findings of a study conducted among university students of Karachi, Pakistan where only 19.3% of the participants were aware of this important information and only 1.3% had undertaken the vaccination [29]. Furthermore, 10% were under the assumption that there was no need to be screened if they were vaccinated, which is comparable to the findings of Khan et al. where 14% were found to be under this false belief [12]. This is largely due to lack of knowledge and the presumption of “not needing it right now“ as found in our study and another study conducted in Karachi [29]. Furthermore, developing countries such as Pakistan are restrained by their limited health budgets; hence, the administration of these vaccines in large-scale populations is difficult because they are cost-intensive. Another common basis for this unsatisfactory practice and the prevalence of insufficient information was the unacceptability and non-recommendation by the physicians as shown in the study conducted in Lahore where health workers' approval played a critical role in influencing the students’ decision to get vaccinated [12].

The attitude regarding undergoing the Pap smear test was found to be negative with only 30.2% willing to be screened in the future. As shown in Table 7, this contrasts remarkably with the study conducted by Ali-Risasi et al., where almost 80% of people were willing to undertake the screening test [25]. However, a study conducted in Pakistan showed similar results in that only one-fourth of the population was willing to go for a screening in the next three years, with the most predictable barrier being the feeling of embarrassment among the females [21]. This depicts that there is a dire need for the creation of a screening program, which will help females become accustomed to these tests without any social barriers. In a study conducted in Indonesia, almost all of the participants were willing to go for HPV vaccination with the most encouraging factor being the belief in the efficacy of the vaccine [24]. However, a study conducted in Karachi showed that only 37.5% were willing to accept the vaccination for themselves, among which almost half of the women believed that the cost of vaccines should be subsidized by the government [30]. These findings are strikingly similar to our study where 49% of women consented to go for vaccination if it was provided free of cost.

Around 80% of women in our study wanted to know more about this disease, its screening, and prevention, which shows a strikingly positive attitude among people towards gaining awareness. However, in a survey conducted by Khan et al., around 93% of women stated that media was not fulfilling its role in raising awareness about this deadly disease and suggested that more information regarding this disease should be made available via campaigns, talk shows, and health bulletins [13]. This shows that a great responsibility lies on the government and health sector of our country in utilizing media as an informative tool for the propagation of awareness regarding cervical cancer, its transmission, prevention, screening, and other relevant information.

Furthermore, there was a significant relationship between knowledge of participants and their socioeconomic status; however, no association was found between socioeconomic status and the willingness of women to undergo Pap smear test and take HPV vaccination. Women from the upper and upper-middle classes had better knowledge than those who belonged to a lower class. This is comparable to a study conducted in India where women with higher household income and those living in urban areas were found to have adequate knowledge [19]. This can be attributed to women with a higher socioeconomic status being able to access media and better healthcare facilities.

Knowledge of the participants regarding cervical cancer was also found to be significantly associated with education, with those with a university-level education having better knowledge scores than the women from other education levels. This is comparable with the findings of two studies conducted in the Maldives and Congo in which women with higher education had increased knowledge [25-26]. A study conducted in Pakistan showed the same association, which signifies that women who are highly educated appear to have access to health information and resources to gain more awareness [21].

The current study also showed that the positive attitude of the females to go for cervical screening and prevention was significantly linked with age, marital status, and education level. A study conducted among Australian women showed a similar correlation: women aged between 30-49 years, who were married, and with higher education were more likely to have had Pap smear tests than their other counterparts [20]. As seen in this study, no correlation was found between attitude and socioeconomic status. A similar relation was established by Lin et al. regarding a higher willingness to get vaccinated with age, higher education levels, and being married [23]. Hereby, it can be concluded that older women who have had more gynecological visits are more aware of malignancies; hence, they are more willing to go for screening tests. Likewise, women who are currently married tend to visit healthcare facilities more frequently and are more prone to have any genital tract infections than those who are not currently in any relationships; thus, they are more open towards screening and preventive measures. Moreover, in Pakistan, there is a cultural trend that the majority of the women, especially from the lower socioeconomic groups, only visit a gynecologist after they are married; therefore, this positive attitude seems to prevail more in that group compared to others.

In a study conducted by Jia et al., higher knowledge scores regarding cervical cancer and a family history of cervical cancer were significantly linked with the willingness to go for screening tests [22]. Likewise, another study conducted in China showed that women who were aware of HPV and its morbidities were more inclined to go for HPV vaccination [23]. The current study found the same correlation between good knowledge scores and a positive attitude to go for screening. This shows that willingness to go for screening and undertaking preventive measures are correlated with higher awareness rates. According to the participants in our study, the most common source of information regarding cervical cancer was family and relatives while doctors, social and print media played comparatively lesser roles. Hence, we feel that social and print media should proactively work in providing information regarding this disease. One such way to do this is by making use of public service messages that are currently commonly employed for breast cancer and family planning awareness. This will help in reducing the stigma surrounding this disease and encourage more women to undertake screening and preventive measures, consequently lessening the burden of this disease. Moreover, 91% of respondents of another research study conducted in Pakistan felt that the government was not fulfilling its role in spreading awareness regarding this disease [13]. Hence, there should be more focus on spreading awareness by the government authorities in order to get favorable outcomes, that is, decreased cervical cancer morbidity and mortality.

The foremost limitation of this study is that it was a single-center study, and we relied on convenient sampling instead of random sampling; hence, the outcomes of this study cannot be generalized to the diverse general population of Karachi. Moreover, due to cultural barriers, this study did not include males who have a pivotal role as family heads in influencing women’s decisions to undertake screening and preventive measures. Future studies should include this population so that an overview at a household level can be obtained. Despite taking a large sample size, this study did not include any rural population. Therefore, future researchers are advised to conduct larger-scale surveys in rural areas where awareness and practices are even more unsatisfactory.

## Conclusions

The findings of this study highlight a dire need for spreading awareness among women of Pakistan regarding HPV and the epidemiology, etiology, screening, and prevention of cervical cancer. Similar to the findings from other studies conducted in Pakistan and other developing countries, unsatisfactory practices and negative attitudes were observed among participants regarding Pap smear screening and HPV vaccination. Furthermore, a significant link was found between knowledge and attitude scores and socioeconomic and demographic parameters. This brings attention to the lack of established national screening programs and large-scale government-subsidized vaccination induction programs in Pakistan, which should be considered and implemented by public and private health sectors in order to decrease the burden of this disease.

## Appendices

Questionnaire

Please note: all responses provided will remain completely anonymous and confidential. By filling this form, you voluntary give consent to be a part of the research "Knowledge, Perception, and Prevention Practices of Human Papillomavirus (HPV) Based Cervical Cancer and its Socioeconomic Correlates Amongst Women in Karachi, Pakistan"

The information provided will be solely used for research purpose(s) only.

A - Sociodemographic profile

1. Age (years)

2. Marital status

o   Single

o   Married

o   Divorced/Separated

o   Widowed

(If single then please skip question no. 3 & 4)

3. Age at marriage (years)

4. No. of children

o   0

o   1-2

o   3-5

o   More than 5

5. Educational status

o   No education

o   Primary school (class 1-5)

o   Secondary school (class 6-10)

o   College

o   University

6. Occupation

o   Student

o   Housewife/Not working

o   Working

o   Jobless/Retired

7. Education of head of the family

o   Professional/Doctorate/Honours/Masters

o   Graduate/Bachelors

o   Intermediate (FSc/FA/A levels) /Diploma

o   High school (Matric/O levels)

o   Middle school (6-8 class)

o   Primary school (1-5 class)

o   No education

8. Occupation of the head of the family

o   Managers/Senior Officials/Legislators

o   Professionals

o   Technicians/Associate or Semi Professionals

o   Clerks

o   Skilled workers/Shop and Market Sales workers

o   Skilled Agricultural/Fishery workers

o   Craft and related trade workers

o   Plant and Machine operators/Assemblers

o   Unskilled workers/Laborers

o   Unemployed

9. Family income per month (PKR)

o   Less than 13,000

o   13,000-39,000

o   40,000-64,000

o   65,000-96,000

o   97,000-129,000

o   130,000-258,000

o   More than 258,000

B - Knowledge questions

B1 - Knowledge about cervical cancer

1. Do you know about the term "cervical cancer"?

o   Yes

o   No

o   Don’t know

   If yes, what is the source of information? (multiple options can be marked)

o   Family/Relatives

o   Friends

o   TV/Radio/Social Media

o   Newspapers/Magazines

o   Doctors

o   Others: Please specify

2. Is cervical cancer one of the most common cancers among females?

o   Yes

o   No

o   Don’t know

3. Are all women at risk of developing cervical cancer?

o   Yes

o   No

o   Don’t know

4. Is cervical cancer more common in middle age (35-50 years) females?

o   Yes

o   No

o   Don’t know

5. Is cervical cancer a communicable disease (transmitted by skin contact, sneezing, coughing)?

o   Yes

o   No

o   Don’t know

B2 - Knowledge about symptoms of cervical cancer

What are the symptoms of cervical cancer from following given options?

1. Lower abdominal pain

o   Yes

o   No

o   Don’t know

2. Bleeding after sexual intercourse

o   Yes

o   No

o   Don’t know

3. Bleeding in between periods

o   Yes

o   No

o   Don’t know

4. Foul smelling vaginal discharge

o   Yes

o   No

o   Don’t know

5. Weight loss

o   Yes

o   No

o   Don’t know

6. Post-menopausal bleeding

o   Yes

o   No

o   Don’t know

7. Asymptomatic (no symptoms)

o   Yes

o   No

o   Don’t know

B3 - Knowledge about risk factors of cervical cancer

What are the risk factors for cervical cancer from following given options?

1. Human papillomavirus (HPV) infection

o   Yes

o   No

o   Don’t know

2. Long term use of oral contraceptives pills

o   Yes

o   No

o   Don’t know

3. Smoking

o   Yes

o   No

o   Don’t know

4. Unprotected sexual practices

o   Yes

o   No

o   Don’t know

5. Multiparity (more than 03 children)

o   Yes

o   No

o   Don’t know

6. Immunocompromised/Human immunodeficiency virus (HIV) - Acquired immunodeficiency syndrome (AIDS)

o   Yes

o   No

o   Don’t know

7. Early age at marriage

o   Yes

o   No

o   Don’t know

B4 - Knowledge about human papillomavirus (HPV)

1. Is HPV infection rare in Pakistan?

o   Yes

o   No

o   Don’t know

2. Can HPV infection affect both males and females?

o   Yes

o   No

o   Don’t know

3. Is HPV transmitted by sexual contact?

o   Yes

o   No

o   Don’t know

4. Can HPV cause cancers other than cervical cancer?

o   Yes

o   No

o   Don’t know

5. Can HPV cause genital warts?

o   Yes

o   No

o   Don’t know

B5 - Knowledge about screening of cervical cancer

1. Is there any test available for screening of cervical cancer?

o   Yes

o   No

o   Don’t know

2. Is pap smear a screening test for cervical cancer?

o   Yes

o   No

o   Don’t know

3. Should screening be initiated at 25 years of age in every female even if asymptomatic (having no symptoms)?

o   Yes

o   No

o   Don’t know

4. Is screening done for sexually active people only?

o   Yes

o   No

o   Don’t know

5. Should screening be repeated every 3 years in every female even if asymptomatic (having no symptoms)?

o   Yes

o   No

o   Don’t know

B6 - Knowledge about prevention of cervical cancer

1. Is HPV vaccine preventable against cervical cancer?

o   Yes

o   No

o   Don’t know

   If yes, what is the source of information? (multiple options can be marked)

o   Family/Relatives

o   Friends

o   TV/Radio/Social Media

o   Newspaper/Magazines

o   Doctors

o   Others: please specify

2. Is HPV vaccine given to males and females both?

o   Yes

o   No

o   Don’t know

3. Should HPV vaccine be given before 26 years of age?

o   Yes

o   No

o   Don’t know

4. Should HPV vaccine be given to sexually active people only?

o   Yes

o   No

o   Don’t know

5. Is there any need for screening even after HPV vaccination?

o   Yes

o   No

o   Don’t know

C - Practices regarding cervical cancer screening and prevention

1. Have you ever had a pap smear done?

o   Yes

o   No

If yes, when is the last time you had pap smear?

o   Less than 1 year

o   Less than 2 years

o   Less than 3 years

o   More than 3 years

   If no, what is the reason? (multiple options can be marked)

o   Expensive

o   Don’t know about the test

o   Time-consuming

o   Embarrassing

o   Parents/spouse won’t allow

o   Might be painful

o   I don’t need it right now

o   Fear of being diagnosed with cancer

o   Others: please specify

2. Are you vaccinated against HPV?

o   Yes

o   No

   If no, what is the reason? (multiple options can be marked)

o   Lack of knowledge

o   Embarrassing in receiving a sexually transmitted infection(STI) vaccine

o   Time-consuming & requires multiple doses

o   Parents/spouse won’t allow

o   Expensive

o   Doubt on vaccine efficacy

o   Worry about side effects

o   Might be painful

o   I don’t need it right now

o   Others: please specify

D - Attitude towards cervical cancer screening and prevention

 (If you have marked yes for any question in the above section please skip the same question number in the section given below)

1. Would you go for a pap smear now?

o   Yes

o   No

2. Do you intend to get vaccinated now?

o   Yes

o   No

   If no, will you consider about getting vaccination if government provides it free of cost?

o   Yes

o   No

3. Would you like to know more about screening and prevention of cervical cancer?

   If yes, what information you would like to know about it? (multiple options can be marked)

o   Who needs it

o   How much it costs

o   Ideal age to get it

o   Methods and duration

o   How effective it is

o   Possible side effects

o   Others: please specify
